# Effectiveness of surgical training in ophthalmology residency programs in Brazil

**DOI:** 10.5935/0004-2749.2025-0033

**Published:** 2025-09-10

**Authors:** Newton Kara-Júnior, Renan Magalhães-e-Silva, Silvana Rossi

**Affiliations:** 1 Department of Ophthalmology, Faculty of Medicine, Universidade de São Paulo, São Paulo, SP, Brazil; 2 Faculty of Medicine, Universidade de São Paulo, São Paulo, SP, Brazil

**Keywords:** Opthalmologists, Teaching, Education, medical, Ophthalmological surgical procedures, Simulation training, Wet lab, Surveys and questionnaires

## Abstract

**Purpose:**

In Brazil, it has traditionally been standard practice to teach a wide range of surgical techniques to all ophthalmology residents, with the aim of equipping them to manage most ocular conditions. However, with modern developments, access to subspecialists has expanded to nearly the entire country. This raises the question of whether it is still necessary to teach numerous surgical techniques to every resident. This study evaluates the effectiveness of surgical training in Brazilian ophthalmology residency programs to determine if comprehensive surgical training for all residents is truly effective, thereby providing evidence to inform educational policy decisions.

**Methods:**

A cross-sectional study using a questionnaire distributed to physicians engaged in eye care.

**Results:**

A total of 137 physicians responded to the survey, with 104 (76.0%) having already completed their specialization. The findings indicate that most practicing ophthalmologists received surgical training during residency in cataract, glaucoma, oculoplastic, and strabismus surgeries. Nonetheless, many of these specialists no longer perform most of these surgeries in practice, except for cataract surgery. While 53.8% of those who completed residency reported satisfaction with their training, 35.6% indicated that they wished they had received better surgical preparation.

**Conclusion:**

The training of ophthalmology specialists must be made more efficient. Training efficiency is reduced when time and resources are devoted to surgical procedures that many specialists will not perform in their careers.

## INTRODUCTION

In Brazil, ophthalmology residency training lasts 3 years and is designed to prepare specialists to clinically and surgically manage most eye diseases. Although this model has recently shifted to a competency-based approach, it has been used in place for three decades and stems from a time when limited communication and transportation options required ophthalmologists to handle all types of ocular conditions locally, often with minimal technological support^(1^,^2)^.

Currently, many ophthalmic surgeries rely on advanced technology and costly equipment that require significant expertise to operate. At the same time, modern developments have improved access to subspecialists throughout almost the entire country. This brings into question whether it remains necessary to train all residents in a broad range of surgical techniques. This issue is even more pertinent given the increasing surgical complexity and the growing number of graduates pursuing fellowships after residency to further develop their surgical competencies^(4^,^5)^.

Many residents may still lack confidence in performing complex procedures independently by the end of their training, which drives them to seek additional subspecialty fellowships for further development. While structural and teaching limitations within training institutions must also be acknowledged, streamlining the residency curriculum to emphasize training in one surgical area per resident could reduce the need for further training and lower the overall time and cost of educating an ophthalmologist^(4)^.

This study set out to assess the effectiveness of surgical training during ophthalmology residency in Brazil, to examine whether instructing all residents in multiple surgical techniques is truly effective, and to generate evidence to inform educational policy.

## METHODS

This cross-sectional study was carried out using a questionnaire distributed to students currently enrolled in ophthalmology residency programs and to physicians who had already completed their specialization. The survey was conducted online from November 24 to December 27, 2024, and only fully completed questionnaires submitted by physicians were included in the analysis.

## RESULTS

A total of 137 physicians completed the survey, of whom 33 (24.0%) were currently enrolled in specialization programs and 104 (76.0%) had already finished their training. Among the graduates, 82 (79.0%) had completed their ophthalmology specialization more than 10 years ago.

Table 1 shows the distribution of surgical procedures offered to the residents still in training. All specialization students who participated reported receiving training in cataract surgery, while about half also received training in oculoplastic and glaucoma surgeries.

**Table 1:** Distribution of surgical procedures available to participants in residency/specialization programs (n=33)

Surgical procedure	n	%
Cataract	33	100.0
Oculoplastic	18	54.5
Corneal suturing	16	48.5
Glaucoma	16	48.5
Strabismus	13	39.4
Refractive	10	30.3
Corneal transplantation	4	12.1
Retina	2	6.0
Orbital	1	3.0

Table 2 displays the distribution of surgical techniques taught during the specialization of those who have already graduated, along with surgical procedures they currently perform in their practice. Most received training in cataract, glaucoma, oculoplastic, and strabismus surgeries during residency. However, many no longer perform most of these surgeries in their current work, except for cataract surgery.

**Table 2:** Comparison of the surgical procedures taught during specialization for graduates and the procedures they currently perform in their practice

Surgical procedure	Currently perform (n=104)	%	Taught during specialization (n=104)	%
Cataract	65	62.5	99	95.2
Corneal suturing	62	59.6	90	86.5
Refractive surgery	47	45.2	20	19.2
Corneal transplantation	37	35.6	50	48.0
Glaucoma	30	28.8	72	69.2
Ocular plastic surgery	22	21.1	56	53.8
Strabismus surgery	12	11.5	57	54.8
Retina	6	5.7	15	14.4
Orbital	4	3.8	10	9.6
None	2	1.9	-	-

Regarding the professional activities of respondents who had completed their training, an average of 48.3% of their time is dedicated to refractometry, 28.3% to the clinical management of eye conditions, and 23.4% to recommending or performing surgeries. While 53.8% of the graduates reported being satisfied with their surgical training, 35.6% stated they would have preferred more robust surgical instruction during residency (Table 3).

**Table 3:** Distribution of graduates based on their perceived satisfaction with their clinical and surgical training

	(n=104)	n (%)
Clinical teaching was lacking	11	10.6
Surgical teaching was lacking	37	35.6
The training was adequate	56	53.8

## DISCUSSION

Is a 3-year residency enough to master multiple surgical techniques safely and effectively? If the training is well-structured and includes solid theoretical and surgical instruction, it may be sufficient. However, without proper planning and support, residents might not feel confident handling complex cases independently and may need to pursue a fellowship of at least two additional years to further develop their skills^(4)^.

However, even in well-designed programs, do all residents actually want to learn multiple surgical techni ques? It appears not. In such situations, the training institution may end up using resources inefficiently, while students spend time learning procedures they are unlikely to perform in practice.

The field of ophthalmology is advancing quickly, yet the training of ophthalmologists still largely follows a model developed in the last century. This makes it necessary to reconsider the foundations of specialist training and adapt them to present-day needs. Much like basic education, where students are grouped by grade levels to ensure consistent content delivery, this standardization approach is also evident in ophthalmology training^(5^-^9)^. Typically, during the first 2 years of residency, students learn how to examine patients and understand eye function, along with performing some surgical procedures. In the third year, they begin to choose specific areas of focus and are taught to perform various surgical techniques. Within this structure, both the duration of training and institutional resources are used to teach all residents to perform multiple surgeries^(10^,^11)^.

This approach generally works well in institutions with sufficient human and material resources, where general ophthalmologists are trained effectively to manage most eye conditions. However, we believe that many training centers cannot provide this level of training adequately.

For this reason, the training process must become more efficient, using the limited human and material teaching resources in a more rational way. Efficiency is lost when time and resources are devoted to surgical training in techniques that professionals are unlikely to use later in their careers. We believe that, after some years of practice, most ophthalmologists who operate will concentrate on one or, at most, two surgical specialties. Today, even in developing countries, good transportation infrastructure across most regions allows both patients and physicians to travel easily, making it unnecessary for every ophthalmologist to be trained in all types of eye surgery^(1)^.

We propose making ophthalmology training more flexible by making surgical training optional and, when chosen, focused on a single area. This would allow residents to perform enough procedures in their chosen field during residency to develop confidence, while freeing up more time to strengthen their general clinical skills.

Institutions that train ophthalmologists have the responsibility to prepare professionals to meet the population’s healthcare needs. Therefore, training should consider not only educational goals but also the broader social context. Given the diversity within the population and the healthcare system, we believe that multiple training pathways should be possible. It is important to discuss a flexible curriculum structure in ophthalmology education so that training can be tailored to each student’s learning needs and aligned with the type of professional they intend to become— whether a generalist, a dedicated subspecialist, or a generalist with a subspecialty focus. Graduates may choose to work as private practitioners in their own clinics, as part of multi-specialist teams, within vertically integrated supplementary health clinics, in diagnostic medicine groups, or in the public health system or pursue careers as teachers, researchers, consultants for the pharmaceutical industry, or innovators, among others^(1)^.

The residency program currently places significant emphasis on surgical training. By streamlining surgical instruction and strengthening general clinical training, resident’s time during specialization could be better utilized, allowing those who do not wish to perform surgeries to devote more time to clinical practice or research. The goal would be for teaching institutions to provide flexible learning pathways, maintaining specialty outpatient clinics with faculty available at all times to address students’ specific interests. This way, there would be a required core curriculum along with optional components, enabling students to choose which clinics to attend and which activities to engage in.

This approach does not mean creating separate clinical or surgical residency tracks. Rather, it aims to let residents customize part of their training during specialization to align with their career goals. In this model, the quantity and type of surgical training each institution offers could be adjusted based on each student’s particular interests.

It is also important to acknowledge that many ophthalmic surgeries have become highly technology-dependent. These advancements have increased the precision and safety of procedures but have also made them more complex to learn. In this context, we believe that it is not enough for teaching hospitals to simply have good infrastructure for patient care, diagnostic exams, and surgeries. Effective surgical training requires more than just skilled professors-it is essential to ensure students have sufficient opportunities for structured theore tical learning. The core of surgical training should take place in the classroom, where techniques and techno logies are explained and surgical videos are discussed. The cognitive aspects of technique and techno logy must be taught in lecture halls rather than relying solely on instruction in the operating room, regardless of the professor’s expertise. Therefore, there must be a strong theoretical training program, ideally led by the surgical instructor, who should apply modern teaching methods. Performing surgery under supervision is the final step of training, where students combine practical manual skills (psychomotor development) with their theoretical understanding of techniques and technology^(11^,^12)^.

We believe that surgical training should follow a clear academic plan so that learning does not depend solely on the student’s own initiative. This approach can help overcome some of the resource limitations of training institutions. Although it may not be feasible to provide every student with an ideal surgical education, a solid training model for each surgical area should include the following elements^(4^,^11)^:

A minimum of 40 hours of theoretical instruction on surgical techniques and related technologies, delivered through active learning methods and taught by qualified instructors.

Required reading of relevant textbooks on the topic.

At least 20 hours of video-based discussions featuring illustrative surgical cases and the management of complications, including sessions where students present videos of surgeries they have performed. We believe more mistakes often teach more than successes, and in a well-documented video, seeing another’s errors is a valuable learning experience.

A minimum of 10 hours of hands-on practice using surgical simulators or wet labs with pig eyes.

After this instructional sequence, the final phase-performing an adequate number of supervised surgeries under the guidance of a surgical mentor-should focus specifically on developing manual skills and applying the technique and technology knowledge already learned.

The optimal period for surgical training is during residency, when learning is the main focus and there is ample access to mentors, patients, equipment, and, above all, time. The sense of having or lacking time can be misleading: during residency, time is abundant and relatively “inexpensive,” but once residency is over, it becomes more limited and “costly.” Therefore, to make the most of this period, it is vital to manage time wisely and use the most effective teaching strategies.

Although this study’s small, non-random sample is a limitation, we believe the findings are sufficient to support the reasoning presented and encourage further investigation on this subject.

In conclusion, the specialist training process must become more efficient. Efficiency is reduced when time and resources are devoted to surgical techniques that the professional is unlikely to perform in their future practice.

## Data Availability

The datasets produced and/or examined in this study can be provided to referees upon request.
